# Bed bugs reproductive life cycle in the clothes of a patient suffering from Alzheimer’s disease results in iron deficiency anemia

**DOI:** 10.1051/parasite/2013018

**Published:** 2013-05-15

**Authors:** Marcela Sabou, Delphine Gallo Imperiale, Emmanuel Andrès, Ahmed Abou-Bacar, Jacinthe Foeglé, Thierry Lavigne, Georges Kaltenbach, Ermanno Candolfi

**Affiliations:** 1 Laboratoire de Parasitologie et de Mycologie Médicale, Hôpitaux Universitaires de Strasbourg 1–3 rue Koeberlé 67000 Strasbourg France; 2 Pôle de Gériatrie, Pavillon Schutzenberger, Hôpital de la Robertsau, Hôpitaux Universitaires de Strasbourg 83 rue Himmerich 67000 Strasbourg France; 3 Médecine Interne Médicale B, Hôpitaux Universitaires de Strasbourg 1 place de l’Hôpital 67000 Strasbourg France; 4 Service d’Hygiène Hospitalière, Hôpitaux Universitaires de Strasbourg 3 rue Koeberlé 67000 Strasbourg France

**Keywords:** Iron deficiency anemia, *Cimex lectularius*

## Abstract

We report the case of an 82-year-old patient, hospitalized for malaise. Her clothes were infested by numerous insects and the entomological analysis identified them as being *Cimex lectularius* (bed bugs). The history of the patient highlighted severe cognitive impairment. The biological assessment initially showed a profound microcytic, aregenerative, iron deficiency anemia. A vitamin B12 deficiency due to pernicious anemia (positive intrinsic factor antibodies) was also highlighted, but this was not enough to explain the anemia without macrocytosis. Laboratory tests, endoscopy and a CT scan eliminated a tumor etiology responsible for occult bleeding. The patient had a mild itchy rash which was linked to the massive colonization by the bed bugs. The *C. lectularius* bite is most often considered benign because it is not a vector of infectious agents. Far from trivial, a massive human colonization by bed bugs may cause such a hematic depletion that severe microcytic anemia may result.

## Abstract (fr)

Nous rapportons le cas d’une patiente âgée de 82 ans, hospitalisée pour malaise. Ses vêtements sont infestés par de multiples insectes et l’analyse entomologique les identifie comme étant des *Cimex lectularius* (punaises des lits). L’anamnèse de la patiente met en exergue des troubles cognitifs sévères. Le bilan somatique retrouve une anémie profonde, microcytaire, arégénerative, fortement ferriprive. Parallèlement, une carence en vitamine B12 imputable à une maladie de Biermer (anticorps anti facteur intrinsèque positifs) est mise en évidence aussi, sans expliquer à elle seule cette anémie sans macrocytose. Un bilan biologique, endoscopique et scannographique élimine une étiologie tumorale responsable d’un saignement occulte. La patiente présente une discrète éruption cutanée prurigineuse, que l’on met en rapport avec la colonisation massive par les punaises des lits. La piqûre de *C. lectularius* est le plus souvent considérée comme bénigne du fait qu’elle n’est vectrice d’aucun agent infectieux. Loin d’être anodine, une colonisation humaine massive par des punaises des lits peut causer une spoliation hématique telle qu’une sévère anémie microcytaire peut en être la conséquence.

## Introduction

Pediculosis and scabies outbreaks are phenomena frequently described in geriatric populations. These are favored by overcrowding in existing institutions and by inadequate knowledge of these pathologies’ diagnosis, often misleading in case of pruritus in the elderly [23]. Colonization by other parasites is less frequent.

We report an isolated case of atypical infestation by an exclusively hematophagous ectoparasite, *Cimex lectularius* [2]. It belongs to the family Cimicidae and is commonly called a “bed bug”. It is an insect known since antiquity [21, 32], which feeds on human blood. It invades places with a high population density where sanitary conditions are poor. Adults measure 4–7 mm, are brownish-red and often compared to apple seeds or lentils. Lucifuge, *C. lectularius* is attracted by body heat and carbon dioxide; it feeds mostly at night, but may also feed during the day if conditions are favorable [2, 4, 20]. The resurgence of bed bugs infestations is a recent phenomenon, probably favored by international trade, immigration, flea markets and garage sales becoming more fashionable, lack of public information, and the changing spectra of resistance to pesticides widely used in the world [2, 18]. *C. lectularius* may also parasitize domestic animals, poultry, birds and bats in the West; migratory birds and bats can be a source of infestation [23, 26, 31]. An emerging ectoparasite and a public health problem in the US [3, 12, 30], Australia [6, 27], Germany [16], Canada [9], Italy [17], Great Britain [24], Korea [13] or France [2, 15], the bed bug has been the subject of guidelines published by the Institutes of Health Surveillance in Europe (European Code of Practice, Bed Bugs Management), US (NPMA BMP Bed Bugs Best Management Practice) or Australia (A code of practice for the Control of Bed Bug Infestations in Australia) [5]. The consequences of their bites are being studied, particularly regarding the potential role in pathogen transmission. Bed bugs have at one time been suspected of being a potential vector of hepatitis B virus [29, 33] or HIV [35]; the latter persists in the saliva of the insect for up to 8 days after the bite, but the transmission of these viruses has not been established [8]. The *C. lectularius* nuisance is mainly due to skin reactions caused by their bites. In highly exposed patients, this may pass virtually unnoticed because of a form of tolerance, and present simply as erythematous maculopapules of 2–5 mm in diameter. Some less exposed subjects may present a severe skin reaction, resulting in a localized urticaria [28]; pruritic papules may later impetiginise. Systemic reactions such as asthma and urticaria with anaphylactic shock have also been described [16]. The immune response to the bed bug bite depends on the host’s immunocompetence status and on the sensitivity to individual salivary components. Thus, patients with urticaria have IgG antibodies directed specifically against certain types of proteins [1]. The presence of skin bullous lesions can be observed and this is due to IgE antibodies directed against nitrophorine, a saliva protein [11, 14].

## Clinical case presentation

We report the case of Mrs R., age 82, addressed to the department of Emergency Medicine after fainting in a public place. The patient was referred to the mobile geriatric unit for cognitive assessment because of a temporo-spatial disorientation, impaired autobiographical memory and a general state of neglect contrasting with good clothes. She was socially isolated and it seemed impossible to find the name of a person or a physician to whom to refer. An emergency CT scan was performed to assess memory impairment, but the results were normal for her age.

Preliminary investigations showed a profound anemia with 5.4 g/dL hemoglobin and 4 μg/L ferritin, with a microcytic and hypochromic aspect, the mean cell volume being low at 67.4 fL. There was no inflammation or fever. Furthermore, clinical examination showed phlebitis in the left leg, which was confirmed by Doppler ultrasonography. There was no exterior bleeding that could explain the anemia. Neurological examination results were within the norm, if not for the presence of cognitive disorders mentioned previously; there was no psychomotor slow down and the thymia was rather neutral. A more careful skin examination revealed the presence of discreet erythematous plaques, apparently not pruritic, in conjunction with confluent maculopapules. The clothes of the patient were colonized by many insects visible to the naked eye, at different stages of development, establishing a true “nest”. Some were still in the nymph stage, flattened and with a translucent body; others reached the size of 7 mm and had a reddish color. The insects were collected and sent to the Parasitology laboratory for entomological analysis, where they were identified as *Cimex lectularius*. This human colonization was somewhat unusual and the Hygiene department of the hospital was contacted in order to determine the details of an isolation protocol. The patient was hospitalized in the Internal Medicine department for assessment and treatment of her anemia. Biologically, iron and ferritin levels were very low, vitamin B9 levels were normal, whereas vitamin B12 was low at 0.14 μg/L. These elements marked the presence of a mixed anemia with both iron and B12 deficiency. In view of its strong microcytic aspect, the predominant mechanism was clearly iron depletion.

The presence of occult bleeding was investigated. Tumor markers were negative. A thoracic-abdominal-pelvic CT searching for neoplasia was also negative. An endoscopic assessment was then performed: the colonoscopy found simple diverticula and the gastroscopy revealed an atrophic gastritis which, associated with the positivity of the intrinsic factor antibodies, was strongly suggestive of pernicious anemia. Joint supplementation with vitamin B12 and iron was started and the anemia was stabilized.

The medical ward where the patient was hospitalized sought geriatric evaluation for a neuropsychological assessment. The Mini Mental state [7] was 11/30 with delirium and forgetting the instructions at the moment of taking the test. The neuropsychological damage was found to be severe and the assessment was not pursued. Severe problems in judgment were highlighted. This assessment, although brief, was performed outside of any mental confusion, when all somatic problems were stabilized and suggested probable diagnosis of Alzheimer’s disease, resulting in memory impairment with altered executive function.

## Pest control measures

When the case was brought to the attention of the Hygiene department by the Parasitology laboratory, many hospital units had already been exposed: an ambulance, a cubicle, one hospital room and two radiology wards. At that stage, early intervention was necessary in order to prevent the infestation of the premises and the proliferation of these insects.

Regarding the ambulance and the emergency cubicle, the environment and mattresses were disinfected after removal of the medical equipment using the Fury^®^ anti-Acariens aerosol insecticide (Spado-Proven Orapi, Villeneuve-Loubet, France), allowed to act for 1 h and followed by a three step cleaning of the premises. The patient’s clothes and all the linen she had been in contact with were put in a clean plastic bag. A-PAR^®^ (Omega-Pharma, Châtillon, France) was added before sealing and marking the plastic bag « bed bugs » . The product was allowed to act for 3 h before sending the bag to be washed separately at a temperature superior to 55 °C.

Concerning the hospital room where the bed bugs had been discovered, the premises have been disinfected using the Fury^®^ anti-Acariens aerosol insecticide, which was applied to all surfaces while the patient herself was being cleaned and given a new hospital gown. The product was allowed to act for 1 h, followed by a three step cleaning.

The two radiology wards were not disinfected, since the patient had already been cleaned and was wearing the new hospital gown before passing through. The imaging department was informed of the situation and of the elements taken into consideration before making this decision.

Once the severe cognitive impairment had been diagnosed, the patient was placed under guardianship. The sanitary department of the city was alerted of the infestation and of the risk of contaminating the neighboring apartments or public transportation, and took the responsibility of the disinfection. The patient’s guardian emptied the apartment and disposed of all of the furniture and of the patient’s belongings; the owner of the apartment was in charge of renovating it. The patient was directly transferred into a nursing home after being released from the hospital.

## Discussion

Classically, bed bug bites are considered benign, because they do not seem to play a role as an infectious vector. This observation calls into question whether bed bugs are really trivial. Indeed, we believe that in the case of our patient, a predominant proportion of anemia was iron deficient, due to its strong microcytic aspect. In the absence of any other etiology (tumor, inflammation, etc) it could have been due to a chronic hematic depletion induced by multiple and repeated bites over a long period of time.

During its existence, each *C. lectularius* female lays 50–500 eggs [10] and the life span is about 10 months at 20 °C. Temperature is the main factor limiting the life cycle. Thus, between 18 and 28 °C, the life span is 120 and 30 days, respectively. The optimal temperature is 28–29 °C and the extreme values beyond which development stops are 15 and 37 °C. The adults are hematophagous for both sexes and feed 2–4 times a week. Each of the five larval transformations present requires a blood meal lasting 5–20 min. Complete development takes 10 weeks at 20 °C. Without feeding, nymphs can survive up to 3 months and adults up to a year or even 2 years in colder environments [2, 4, 20, 23]. In the case of our patient, due to the precarious hygiene conditions, the developmental cycle probably took place in the clothes from which she almost never parted. The volume of blood ingested is equal, on average, to 7 mm^3^ of blood [19] and it has been shown that at controlled temperatures of 32 °C bed bugs refeed every 1–3 days [25]. This would explain the development of iron deficiency anemia in this particular case, where the insects probably lived in the clothes of the patient.

The anemia observed in our case was extremely severe. Pernicious anemia was obviously involved, but iron deficiency clearly played an important role, with a net impact on the profile of anemia. This is the fourth case reported in the literature where iron deficiency anemia appears to be due to repeated bed bug bites [22, 23, 34]. Because of the resurgence of bed bugs, this is a diagnosis not to be ignored, notably among our elderly patients and especially those with a cognitive deficit. Indeed, in this observation, the existence of an Alzheimer’s disease was clearly an aggravating factor. The impaired judgment, loss of memory and the resulting social isolation, clearly favored the abnormal expansion and persistence of this parasite. This case is unique because of the establishment of a true bed bug “nest” in the patient’s clothes.

Bed bugs quickly nest in remote areas, most often behind frames, power strips and in folds of mattresses. This invasion is facilitated by their mobility and their ability to survive in hostile environments. In our case, the situation was even more unusual because of the large amount of insects brought into the hospital environment.

Infestations with bed bugs are difficult to control because of their behavior and resistance to commonly used pesticides [20]. The pest control described recently is that of Integrated Pest management [5, 20]. It consists in correctly identifying the insect, determining the affected areas and in combining non-chemical control techniques with insecticide use. Specially trained dogs can detect affected areas and vacuuming can reduce the number of parasites.

Infested clothes, linens and pillows should be placed in sealed plastic bags; the use of extreme temperatures (over 45–60 °C or below 17 °C) can kill *C. lectularius*. Mattresses can be vacuumed and steamed or treated with various pesticides [20]. Choice of pesticides is critical and should be made wisely because of acquired resistance and according to the area or objects that are being treated. Dust products seem to be more effective than aerosols or sprays; fumigants seem to have the best penetrability but are highly toxic for humans [5]. In the medical setting, affected rooms should quickly be isolated and disinfected. Adjoining rooms should also be inspected. Exposure of other patients or health care workers to bed bugs should be investigated [20].

Regarding the pest control measures taken in our case, treating the patient’s clothes and bed linens with an insecticide before sending them to be washed might seem overzealous. Simply sealing these articles in a plastic bag and freezing them or having them washed separately at temperatures over 55 °C would have sufficed. Choosing the right insecticide to treat the affected rooms is not an easy decision. Other medical institutions confronted to bed bugs infestations have used new generation pyrethroids or apyrrole insecticides in order to avoid acquired resistance problems [5, 22]. Spread of the infestation is controlled by rapid isolation and treatment of the premises. These measures can be cumbersome to implement depending on affected units.

## Conclusions

This case reinforces the idea that *C. lectularius* infestation can have serious somatic and institutional consequences.

Indeed, this is the fourth case described in the literature where repeated bed bug bites have caused such a hematic depletion, that severe iron deficiency anemia was the consequence [22, 23, 34]. This is a diagnosis not to be ignored in the current resurgence of bed bug infestations, either in the case of population migrations or of unsanitary conditions. The geriatric population represents a growing demographic and is a target population that appeared unaffected by this parasite threat in the past. However, social isolation caused by cognitive impairment and Alzheimer’s disease was an aggravating factor to consider in the case of our patient, allowing for an unusual expansion of the parasite in the patient’s clothes.

Entomological analysis, fast and efficient collaboration between the departments of parasitology, hygiene and medicine have helped define and contain the colonization by *C. lectularius*. Parasite dissemination in our institution would have resulted in quarantined beds making access to care difficult.
